# Supplementary material for: Calanus Oil and Lifestyle Interventions Improve Glucose Homeostasis in Obese Subjects with Insulin Resistance
Source: Mar Drugs. 2025 Mar 23;23(4):139. doi: 10.3390/md23040139 (PMC12028837; doi:10.3390/md23040139)
Supplement: Supplementary file 1 [file marinedrugs-23-00139-s001.zip › marinedrugs-3513725-supplementary.pdf]

Supplementary

Table S1: Parameters of the 6-minute walk test before (t_0) and after (t_{12}) the intervention.

		2 g CO/day		4 g CO/day		2 g CO/day + LI		Placebo	p-value
**Variables**	n	Mean ± SD	n	Mean ± SD	n	Mean ± SD	n	Mean ± SD	
**Pulse rate** before the test [per min]									
t_0	61	81.5±14.4*	61	73.9±12.1	57	78.2±13.5	58	75.7±11.6	**0.011**[a]
t_{12}	61	77.6±13.2	61	75.0±13.3	57	77.3±13.7	58	76.5±15.8	0.781[a]
Δt_{12}- t_0	61	-4.0±11.9	61	0.2±10.1	57	0.7±10.4	58	0.3±12.0	0.109[b]
**SpO_2** before the test [%]									
t_0	61	97.1±1.2	61	96.3±1.8	57	95.7±5.1	58	96.4±1.6	0.087[a]
t_{12}	61	96.7±1.7	61	95.7±3.1	57	95.9±4.8	58	96.2±1.8	0.382[a]
Δt_{12}- t_0	61	-0.5±2.0	61	-0.7±2.9	57	0.2±7.1	58	-0.2±2.1	0.708[b]
**Distance** [m]									
t_0	61	539±61.3	61	524±86.2	57	533±72.2	58	535±70.8	0.722[a]
t_{12}	61	548±59.2	61	527±84.6	57	555±64.5	58	544±64.4	0.209[a]
Δt_{12}- t_0	61	7.2±55.5	61	5.0±35.0	57	13.7±40.7	58	6.4±56.6	0.814[b]

Abbreviations: CO, Calanus oil; LI, lifestyle intervention

Significant p-values ($p<0.05$) are shown in bold.

[a] One-way ANOVA was used to determine the difference in parameters of 6-minute walk test (6MWT) before (t_0) and after the intervention (t_{12}).

[b] ANCOVA with change in parameters of 6MWT (Δt_{12}- t_0) as dependent variable, study group as a fixed factor and BMI, and age as covariates.

Significant difference in post hoc tests: * 2 g CO/day vs 4 g CO/day and 2 g CO/day vs Placebo.

Table S2: Parameters of regular physical activity before (t_0) and after (t_{12}) the intervention.

		2 g CO/day		4 g CO/day		2 g CO/day + LI		Placebo	p-value
**Variables**	n	Mean ± SD	n	Mean ± SD	n	Mean ± SD	n	Mean ± SD	
**Basic activity** [h/w]									
t_0	61	5.9±6.6	61	5.4±5.4	57	5.1±5.8	58	5.4±5.6	0.903[a]
t_{12}	61	9.2±9.0	61	8.2±6.8	57	6.1±5.6	58	7.5±5.8	0.104[a]
Δt_{12}- t_0	61	2.6±8.8	61	3.3±3.0	57	1.4±6.4	58	1.9±5.6	0.109[b]
**Sport activity** [h/w]									
t_0	61	2.5±3.4	61	1.9±2.5	57	1.5±2.2	58	1.3±2.2	0.055[a]
t_{12}	61	3.2±4.3	61	2.7±4.4	57	2.9±4.1	58	2.5±3.6	0.806[a]
Δt_{12}- t_0	61	0.7±2.9	61	0.8±3.5	57	1.3±2.9	58	0.9±3.5	0.551[b]
**Leisure activity** [h/w]									
t_0	61	3.6±4.5	61	2.6±3.1	57	2.3±2.4	58	2.9±3.1	0.157[a]
t_{12}	61	3.4±3.7	61	2.7±5.7	57	4.2±5.8	58	2.8±3.0	0.300[a]
Δt_{12}- t_0	61	-0.3±4.7	61	0.3±5.8	57	2.0±6.5	58	0.1±2.5	0.053[b]
**Sleep duration** [h/w]									
t_0	61	6.8±1.0	58	7.0±1.0	57	6.9±1.1	58	6.8±1.1	0.717[a]
t_{12}	55	6.9±1.0	54	6.9±1.0	57	6.8±0.9	58	6.8±1.1	0.944[a]
Δt_{12}- t_0	55	0.1±1.0	54	-0.1±0.8	57	-0.2±1.0	58	0.0±0.9	0.570[b]

Abbreviations: CO, Calanus oil; h/w, hour per week; LI, lifestyle intervention

Significant p-values ($p<0.05$) are shown in bold.

[a] One-way ANOVA was used to determine the difference in parameter of regular physical activity (t_0). and after the intervention (t_{12}).

[b] ANCOVA with change (Δt_{12}- t_0) in parameters of regular physical activity as dependent variable, study group as a fixed factor and BMI, and age as covariates.

Table S3: Parameters of body composition (t_0) and after (t_{12}) the intervention.

	2 g CO/day		4 g CO/day		2 g CO/day + LI		Placebo		p-value
**Variables**	n	Mean ± SD	n	Mean ± SD	n	Mean ± SD	n	Mean ± SD	
**Phase angle** [°]									
t_0	61	6.0±0.8	61	5.8±0.8	57	5.9±0.7	58	5.8±0.7	0.232[a]
t_{12}	61	6.0±0.7	61	5.9±0.9	57	5.9±0.8	58	5.9±0.8	0.928[a]
Δt_{12}- t_0	61	-0.1±0.6	61	0.1±0.4	57	0.0±0.5	58	0.1±0.5	0.278[b]
**FM** [%]									
t_0	61	42.5±7.4	61	42.9±7.6	57	41.8±7.9	58	42.8±7.6	0.246[a]
t_{12}	61	42.2±7.7	61	41.7±7.2	57	42.1±8.4	58	42.5±8.0	0.928[a]
Δt_{12}- t_0	61	-0.7±5.4	61	-1.6±3.6	57	0.1±3.3	58	-0.6±3.6	0.238[b]
**Visceral FM** [l]									
t_0	61	4.3±2.5	61	4.3±2.1	57	4.3±2.7	58	4.2±2.2	0.997[a]
t_{12}	55	4.5±3.0	61	4.0±2.0	57	4.3±2.6	58	4.2±2.2	0.825[a]
Δt_{12}- t_0	55	0.2±1.8	61	-0.3±1.2	57	0.1±1.0	58	0.0±0.9	0.286[b]

Abbreviations: CO, Calanus oil; FM, fat mass; l, liter; LI, lifestyle intervention

Significant p-values ($p<0.05$) are shown in bold.

[a] One-way ANOVA was used to determine the difference in parameter of body composition (t_0). and after the intervention (t_{12}).

[b] ANCOVA with change (Δt_{12}- t_0) in parameters of body composition as dependent variable, study group as a fixed factor and BMI, and age as covariates.

Table S4: Study characteristics at baseline (n=266) (study cohort as intention to treat).

	2 g CO/day n=69	4 g CO/day n=67	2 g CO/day + LI n=68	Placebo n=62	*p-value*
**Variables**	n (%)	n (%)	n (%)	n (%)	
**Gender**					
Female	48 (70)	46 (69)	48 (71)	41 (66)	0.955[a]
Male	21 (30)	21 (31)	20 (29)	21 (34)	
**Anthropometric**	Mean ± SD	Mean ± SD	Mean ± SD	Mean ± SD	
Age [y]	53.8±9.9	57.3±11.3	54.3±10.2	54.7±9.4	0.324[b]
BMI [kg/m^2]	34.4±5.4	34.6±4.3	35.2±6.9	34.3±4.4	0.903[b]
WC [cm]	110±12.0	111±10.7	111±13.9	111±11.0	0.759[b]
**Body composition**					
Phase angle [°]	6.0±0.7	5.8±0.8	6.0±0.7	5.8±0.7	0.235[b]
Body fat [%]	42.8±7.1	42.7±7.6	42.0±7.9	43.1±8.0	0.883[b]
Visceral fat mass [l]	4.1±2.4	4.3±2.2	4.4±2.8	4.1±2.2	0.897[b]
**Metabolic Syndrome Severity (MetS) Score**					
MetS score (based on BMI)	0.6±0.6	0.6±0.5	0.6±0.7	0.5±0.5	0.884[b]
MetS score (based on WC)	0.6±0.8	0.6±0.5	0.6±0.6	0.5±0.5	0.760[b]

Abbreviations: BMI, body mass index; CO, Calanus oil; LI, Lifestyle intervention; SD, standard deviation; WC, waist circumference.

[a] chi-squared test. [b] one-way ANOVA.

Table S5: Concentration of fasting Insulin and fasting Glucose before t_0 and after t_{12} the intervention.

	2 g CO/day	4 g CO/day	2 g CO/day + LI	Placebo	p-value		Effect size
	n=61	n=61	n=57	n=58			
**Variables**	**Mean±SD**				**At t_0[a]**	**Inter-ation[b]**	**η_p^2**
**Insulin** [µU/L]							
t_0	15.0±7.0	15.5±8.0	15.1±7.2	14.9±6.4	0.953		
t_{12}	15.3±7.8	15.4±9.7	14.8±7.5	15.1±7.7		**0.006****	0.048
**Glucose** [mg/dl]							
t_0	103±9.1	103±7.7	106±8.1	104±8.7	0.584		
t_{12}	104±9.0	103±8.2	104±11.1	104±11.3		**0.002****	0.074

Abbreviations: AU, arbitrary unit; CO, Calanus oil; HOMA index, homeostatic model assessment of insulin resistance; LI, lifestyle intervention; SD, standard deviation; η_p^2 partial eta square.

Significant p-values ($p<0.05$) are shown in bold.

[a] Difference before the intervention at t_0: One-way ANOVA.

[b] ANCOVA with change in insulin; glucose (Δt_{12}- t_0) as dependent variable, study group as a fixed factor and centered Variables at t_0, squared Variables at t_0, BMI, age and sex as covariates.

Significant difference in post hoc tests: * Group 4 g CO/day vs Placebo group; **Group 2 g CO/day + LI vs Placebo group.
